# Zn^2+^-induced changes at the root level account for the increased tolerance of acclimated tobacco plants

**DOI:** 10.1093/jxb/eru251

**Published:** 2014-06-13

**Authors:** Nadia Bazihizina, Cosimo Taiti, Lucia Marti, Ana Rodrigo-Moreno, Francesco Spinelli, Cristiana Giordano, Stefania Caparrotta, Massimo Gori, Elisa Azzarello, Stefano Mancuso

**Affiliations:** ^1^LINV - Department of Agrifood Production and Environmental Sciences – University of Florence, Viale delle Idee 30, 50019 Sesto F.no, Florence, Italy; ^2^Centro di Microscopie Elettroniche ‘Laura Bonzi’ (Ce.M.E.), ICCOM, CNR, Via Madonna del Piano, 50019 Sesto F.no, Florence, Italy

**Keywords:** Acclimation, heavy-metal toxicity, membrane potential, *Nicotiana tabacum*, stomatal conductance, transporter, vacuole.

## Abstract

Evidence suggests that heavy-metal tolerance can be induced in plants following pre-treatment with non-toxic metal concentrations, but the results are still controversial. In the present study, tobacco plants were exposed to increasing Zn^2+^ concentrations (up to 250 and/or 500 μM ZnSO_4_) with or without a 1-week acclimation period with 30 μM ZnSO_4_. Elevated Zn^2+^ was highly toxic for plants, and after 3 weeks of treatments there was a marked (≥50%) decline in plant growth in non-acclimated plants. Plant acclimation, on the other hand, increased plant dry mass and leaf area up to 1.6-fold compared with non-acclimated ones. In non-acclimated plants, the addition of 250 μM ZnSO_4_ led to transient membrane depolarization and stomatal closure within 24h from the addition of the stress; by contrast, the acclimation process was associated with an improved stomatal regulation and a superior ability to maintain a negative root membrane potential, with values on average 37% more negative compared with non-acclimated plants. The different response at the plasma-membrane level between acclimated and non-acclimated plants was associated with an enhanced vacuolar Zn^2+^ sequestration and up to 2-fold higher expression of the tobacco orthologue of the *Arabidopsis thaliana MTP1* gene. Thus, the acclimation process elicited specific detoxification mechanisms in roots that enhanced Zn^2+^ compartmentalization in vacuoles, thereby improving root membrane functionality and stomatal regulation in leaves following elevated Zn^2+^ stress.

## Summary

Exposing plants to non-toxic metal concentrations elicits specific detoxification mechanisms in tobacco roots that improve root membrane functionality and leaf stomatal regulation with toxic zinc in the growing medium.

## Introduction

Zinc releases into the environment are associated with biotic or natural atmospheric processes, but mining and anthropic activities have resulted in heavy-metal contamination of urban and agricultural soils (Friedland, 1990). Furthermore, an increasing acidity of soils liberates the bound pool of metals, which in turn leads to increased availability of metal ions for plants (Påhlsson, 1989). Zn^2+^ is crucial for the metabolism of plant cells, being involved in a wide variety of physiological processes at the micromolar range; however, Zn^2+^ is toxic to plants at supra-optimal concentrations, and toxicity occurs when leaf concentrations reach 400–500 μg g^–1^ of dry mass (Marschner, 1995; Broadley *et al.*, 2007). Common Zn^2+^ toxicity symptoms include: reduced plant water content and stunted plant growth (Sagardoy *et al.*, 2009), decreased stomatal conductance and photosynthesis (Sagardoy *et al.*, 2010), changes in root growth and morphology, severe nutrient imbalances, and leaf chlorosis (Marschner, 1995; Vaillant *et al.*, 2005; Broadley *et al.*, 2007; Sagardoy *et al.*, 2009).

Acclimation occurs during plant ontogeny and describes the enhanced stress tolerance of a particular individual plant as a result of the induction of physiological, biochemical, and molecular adjustments within the plant’s tissues and cells (Pandolfi *et al.*, 2012). Plant acclimation to a particular abiotic stress condition is associated with responses tailored to the specific conditions encountered (Mittler, 2006). There is some evidence suggesting that heavy-metal tolerance can be induced in plants following a pre-treatment (i.e. acclimation) with non-toxic metal concentrations, which improve the plant’s ability to tolerate otherwise toxic metal concentrations (Watmough and Dickinson, 1996). For example, heavy-metal resistance traits (e.g. reduced growth inhibition in response to heavy metal) were induced in a cell suspension culture from shoot explants of mature trees of *Acer pseudoplatanus* through repeated exposure to gradually increasing metal concentration in the growth medium (Dickinson *et al.*, 1992). Nevertheless, plant acclimation to heavy metals remains a controversial topic in the literature, and currently little information is available regarding the eventual mechanism(s) underlying the increased tolerance to toxic heavy-metal concentrations in acclimated plants (e.g. Turner and Dickinson, 1993; Wisniewski and Dickinson, 2003). By contrast, other examples of acclimation processes in plants, such as cold acclimation (i.e. increased freezing tolerance following exposure to low non-freezing temperatures; Thomashow, 1999) or the increased ability of plants to tolerate toxic salt concentrations after exposure to non-toxic salinities (Silveira *et al.*, 2001; Djanaguiraman *et al.*, 2006; Pandolfi *et al.*, 2012) are well accepted.

Metal ions such as Cd^2+^ and Zn^2+^ have been found to induce serious and continuous membrane depolarization in root cells (Kennedy and Gonsalves, 1987; Aidid and Okamoto, 1992). As the plant plasma membrane and its functions have been regarded as the first targets of heavy-metal toxicity, any form of tolerance should involve protection of membrane integrity (Hall, 2002). In support of this hypothesis, the plasma membrane of tolerant plants generally experiences less metal-induced damage than that of sensitive plants (Kenderesová *et al.*, 2012). Tolerance to high levels of heavy metals is associated with sequestration of ions in metabolically inactive compartments (i.e. vacuoles) (Hall, 2002; Krämer *et al.*, 2007), and the presence of active cytoplasmic Zn^2+^ has been found to induce plasma-membrane depolarization (Kenderesová *et al.*, 2012); it could therefore be hypothesized that, in acclimated plants, prior exposure to non-toxic Zn^2+^ concentrations will induce specific detoxification mechanisms that, following the addition of elevated and toxic Zn^2+^ concentrations, will reduce the build-up of Zn^2+^ in sensitive and metabolically active sites of the cell and ultimately result in an improved root membrane functionality.

Zinc toxicity inhibits both photosynthesis and stomatal conductance (Sagardoy *et al.*, 2009; Azzarello *et al.*, 2012). However, it is still unclear whether photosynthesis inhibition or a perturbation of the water, and thus stomatal limitations, are one of the primary causes of heavy-metal toxicity (including Zn^2+^ toxicity) at the shoot level (Perfus-Barbeoch *et al.*, 2002; Sagardoy *et al.*, 2010). Increasing evidence suggests that exposure to toxic metal concentrations negatively affects parameters important for plant–water relationships, and, in particular, toxic metal has been found to reduce the biomass allocation to the roots (Ryser and Emerson, 2007) reduce cell-wall elasticity (Barceló *et al.*, 1986) increase cell-membrane permeability (Llamas *et al.*, 2008; Michael and Krishnaswamy, 2011); reduce stem and root hydraulic conductivity (Przedpelska-Wasowicz and Wierzbicka, 2011; de Silva *et al.*, 2012), and reduce xylem-specific and leaf-specific hydraulic conductivity (de Silva *et al.*, 2012). Hence, given that perturbation of leaf stomatal regulation (Sagardoy *et al.*, 2010) has been considered one of the early causes of heavy-metal toxicity (e.g. within the first 48h after treatment; Sagardoy *et al.*, 2010), it is conceivable that the acclimation process will result in an improved stomatal regulation upon exposure to heavy-metal stress.

In the present study, we tested whether pre-treatment for 1 week with a high but non-toxic Zn^2+^ concentration had any effect on the tolerance of tobacco (*Nicotiana tabacum*) to toxic Zn^2+^ concentrations. We hypothesized that the acclimation process would substantially decrease the symptoms generally associated with Zn^2+^ toxicity, and would therefore increase: (i) shoot and root growth, and (ii) the total chlorophyll and carotenoid concentrations in leaves. In addition, considering that plant acclimation requires responses tailored to the specific external environmental conditions (Mittler, 2006), we hypothesized that, following Zn^2+^ addition, the acclimation process to Zn^2+^ would: (iii) improve stomatal regulation, and (iv) result in a superior ability to maintain negative membrane potential in roots, as these are the first organs to encounter the heavy-metal stress. We also expected that this improved root membrane functionality would be associated with (iv) an enhanced sequestration of Zn^2+^ in the vacuole.

## Materials and methods

### Plant material and growth conditions

Tobacco plants (*N. tabacum*) were germinated and grown in environmentally controlled chambers (25/25 °C day/night, 12h day/12h night, with an average photosynthetically active radiation at shoot height of 300 µmol m^–2^ s^–1^). Seeds of tobacco were sown in plastic pots containing standard potting mix, and 2 (Experiments 2 and 3) or 5 (Experiment 1) weeks after emergence, seedlings were transferred to an aerated nutrient solution. All plants were supplied with half-strength Hoagland’s nutrient solution (pH adjusted to 5.8 using KOH). The pH of the solution was checked and adjusted (as required) daily and solutions were changed weekly. Two weeks after transferring the plants to the aerated nutrient solution, ZnSO_4_ was added to the aerated solutions to obtain the required final Zn^2+^ concentrations.

### Experimental design

#### Responses of tobacco to elevated ZnSO_4_ in three different experiments. 

Experiment 1 consisted of eight treatments with four replicates in a completely randomized block design. In six treatments, plants were exposed to increasing Zn^2+^ concentrations: 1 μM ZnSO_4_, considered as the control treatment, and three other treatments where the appropriate amount of ZnSO_4_ was added to the control solution to reach final concentrations of 30, 250, and 500 μM ZnSO_4_. In the remaining two treatments, 1 week prior to the treatment (250 μM ZnSO_4_), plants were exposed to a high but non-toxic Zn^2+^ concentration of 30 μM ZnSO_4_ (cf. Arrivault *et al.*, 2006). Plants were then harvested 3 weeks after imposing the treatments. To elucidate the possible mechanism(s) responsible for the enhanced tolerance in acclimated plants, two additional experiments (Experiments 2 and 3) were conducted focusing on the responses in the short-term (within 24h) to 250 μM ZnSO_4_. In these two experiments we tested whether, following the addition of 250 μM ZnSO_4_, the acclimation process was associated, at the root level, with an improved ability to maintain negative membrane potentials and sequestrate Zn^2+^ in the vacuoles. The experiments consisted of three treatments with four replicates in a randomized block design. In two treatments, plants were exposed to two Zn^2+^ concentrations (1 and 250 μM ZnSO_4_), and in the remaining treatment, 1 week prior to the addition of 250 μM ZnSO_4_, plants were exposed to 30 μM ZnSO_4_.

#### Plant sampling (Experiment 1) 

Plants were sampled 20 d after applying the treatments for the determination of shoot and root fresh and dry masses. Shoot and root tissues were harvested and their fresh weight recorded. Leaves were scanned for surface area and leaf area calculated using the Tomato Analyzer software. In addition, root samples were taken for subsequent transmission electron and light microscopy. Shoot and roots were then oven dried at 60 °C to determine their dry mass. In addition, plants were sampled before applying the treatments and then 24h after the commencement of treatments. Shoot and root tissues were harvested and their fresh mass recorded. Shoot and roots were then oven dried at 60 °C to determine their dry mass. Plant fresh and dry masses were used to calculate the plant water content (WC) on a fresh weight basis using the following equation: WC (%)=[(fresh mass – dry mass)/fresh mass]×100.

#### Leaf pigment analyses (Experiment 1) 

In Experiment 1, at the end of the experimental period, total chlorophyll and carotenoid concentrations were determined in all treatments by reading the absorbance at 537, 647, and 664nm of extracts obtained from two disks of 10mm in diameter taken from randomly selected youngest fully expanded leaves from each replicate. Leaf discs were ground in liquid nitrogen and extracted with an acetone and Tris buffer solution for 48h at 4 °C in the dark (Sims and Gammon, 2002). Chlorophyll and carotenoid concentrations were determined according to Wellburn and Lichtenthaler (1984) using a Tecan Infinite 200 Spectrophotometer (Männedorf, Switzerland).

#### Transmission electron microscopy (Experiment 1) 

Samples of the control and ZnSO_4_-treated roots were cut into pieces 3mm long and immediately fixed in 2.5 % glutaraldehyde in 0.2M phosphate buffer (pH 7.2) for 2h at room temperature. Samples were then washed twice in the same buffer and post-fixed in 2% OsO_4_ in the same buffer for 2h at room temperature. Following dehydration in a graded ethanol series (30, 40, 50, 60, 70, 80, 95, and 100%), the specimens were gradually embedded in Spurr resin (Spurr, 1969) and polymerized at 70 °C for 24h. Ultrathin (70–90nm) transverse sections of the processed tissue were obtained with an LKB IV ultramicrotome, collected on Formvar-coated aluminium grids, stained with uranyl acetate and lead citrate, and examined using a Philips CM12 transmission electron microscope (Eindhoven, The Netherlands) operating at 80kV.

#### Light microscopy (Experiment 1) 

The root tissue was processed and cut as for transmission microscopy. Semi-thin sections of 1–2 μm were fixed to glass slides, and observations were carried out in a Leica DM LB2 Light Microscope (Leica Microsystems Wetzlar GmbH, Germany).

#### Membrane potential measurements (Experiment 2) 

Membrane potentials were measured on cortical cells of excised root segments of plants grown in aerated solution with 1 μM (control conditions) or 30 μM (acclimated plants) ZnSO_4_. Excised root segments were immobilized in a Plexiglass chamber filled with 4ml of buffered Tris/MES basal salt medium (BSM: 0.2mM KCl, 0.1mM CaCl_2_, pH 5.8) for 4h before the measurements. Cells were impaled with conventional KCl-filled Ag/AgCl microelectrodes (Shabala and Lew, 2002; Cuin and Shabala, 2005), and membrane potentials were recorded for 2min. Subsequently 1ml of buffered Tris/MES BSM with 1.25mM ZnSO_4_ was added, resulting in a final concentration of 250 μM ZnSO_4_ in the bath solution. Measurements were continued for another 10min after addition of the ZnSO_4_ solution. Four individual plants for each treatment were measured. Subsequently membrane potentials were measured in treated plants (250 μM ZnSO_4_) after 24h of treatment. Four individual plants for each treatment were measured, with up to five readings from each individual root.

#### Leaf gas-exchange parameters (Experiment 2) 

Leaf gas-exchange parameters were determined simultaneously with chlorophyll fluorescence measurements using the open gas-exchange system Li-6400 XT (Li-Cor, Lincoln, NE, USA) with an integrated fluorescence chamber head (Li-6400–40; Li-Cor). Leaf gas-exchange measurements were taken on all plants in each treatment, before the treatment (0h) and 24h after adding 250 μM ZnSO_4_. Measurements of net photosynthetic rate (*A*_n_), stomatal conductance (*g*_s_), and substomatal CO_2_ concentrations (*C*_i_) were determined on the youngest fully expanded leaves at ambient relative humidity (40–50%), reference CO_2_ of 400 µmol mol^–1^, flow rate of 400 µmol s^–1^, chamber temperature of 25 °C and photosynthetically active radiation of 300 µmol m^–2^ s^–1^.

Using the integrated fluorescence chamber head (Li-6400–40) of the open gas-exchange system Li-6400 XT, we measured chlorophyll fluorescence on the same leaves used for gas-exchange measurements at the end of the night period (i.e. when plants had been in the dark for at least 11h, before the lights were switched on in the controlled environment room). The minimal fluorescence level in the dark-adapted state (*F*_0_) was measured using a modulated pulse, and maximal fluorescence in this state (F_m_) was measured after applying a saturating actinic light pulse of 7000 μmol m^–2^ s^–1^. The values of the variable fluorescence (*F*_v_=*F*_m_–*F*_0_) and maximum quantum efficiency of photosystem II (PSII) photochemistry (F_v_/F_m_) were calculated from *F*_0_ and *F*_m_.

#### Confocal microscopy (Experiment 3) 

Confocal imaging was performed using an upright Leica laser-scanning confocal microscope SP5 (Leica Microsystems Wetzlar GmbH, Germany) equipped with a 40× oil-immersion objective. To analyse the intracellular localization of the Zn^2+^ ions in root cells, FluoZin-3-AM (acetoxymethyl) cell permeant (Molecular Probes, USA) was used. FluoZin-3-AM was chosen as it is considered to be a very specific indicator for intracellular Zn^2+^ localization and concentration (Gee *et al.*, 2002). Roots were incubated for 60min in a solution of 15 µM FluoZin-3-AM. After incubation, the samples were mounted in a water solution on a slide and observed. The excitation wavelength was set at 488nm, and emission was detected at 530±20nm.

#### Expression of Zn^2+^ metal tolerance protein 1 (MTP1) protein transporter in root tissues (Experiment 3) 

After 24h of treatments, roots were collected from tobacco seedlings and immediately frozen in liquid nitrogen. Samples were homogenized with a pestle and total RNA was extracted with an RNeasy Plant Mini kit (Qiagen). First-strands cDNA was synthesized using a Quantitect Reverse Transcription kit (Qiagen) according to the manufacturer’s instructions. The quantity of RNA and cDNA was measured using a Tecan Infinite 200 Spectrophotometer (Männedorf, Switzerland). Transcript levels were determined by quantitative real-time PCR (RT-qPCR) with a QuantiFast SYBR Green PCR kit (Qiagen) using a Rotor-Gene 6000 (Corbett Life Science). RT-qPCR was conducted in a 15 μl reaction mixture volume and the protocol was: initial step of 95 °C for 5min, and 40 cycles of 95 °C for 12 s and 60 °C for 45 s, followed by meltin-curve analysis. Each sample, standard curve and no-template control were run in triplicate. The primer sequences for the target gene were designed in a common region for the tobacco orthologue of the *Arabidopsis thaliana MTP1* gene (*NtMTP1a* and *NtMTP1b* genes; for more details, see Shingu *et al.*, 2005). As housekeeping genes, *EF-1α* and *L25* were used, as these have been found to have the highest stability under abiotic stresses in tobacco (Schmidt and Delaney, 2010). As results were similar for the two housekeeping genes, only *EF-1α* data are shown. Primer sets used for RT-qPCR are listed in Table S1 at *JXB* online. Relative expression data were calculated using the comparative Livak method (2^–ΔΔCT^; Livak and Schmittgen, 2001). The target fragment was verified by sequencing.

### Statistical analyses

Statistical analyses were conducted using GraphPad for Mac, 6th edn. One-way or two-way analysis of variance, depending on the dataset, was used to identify overall significant differences between treatments. Unless otherwise stated, the significance level was *P≤*0.05.

## Results

### A 1-week acclimation period with 30 μM ZnSO_4_ increases Zn^2+^ tolerance in the long term

**Fig. 1.**
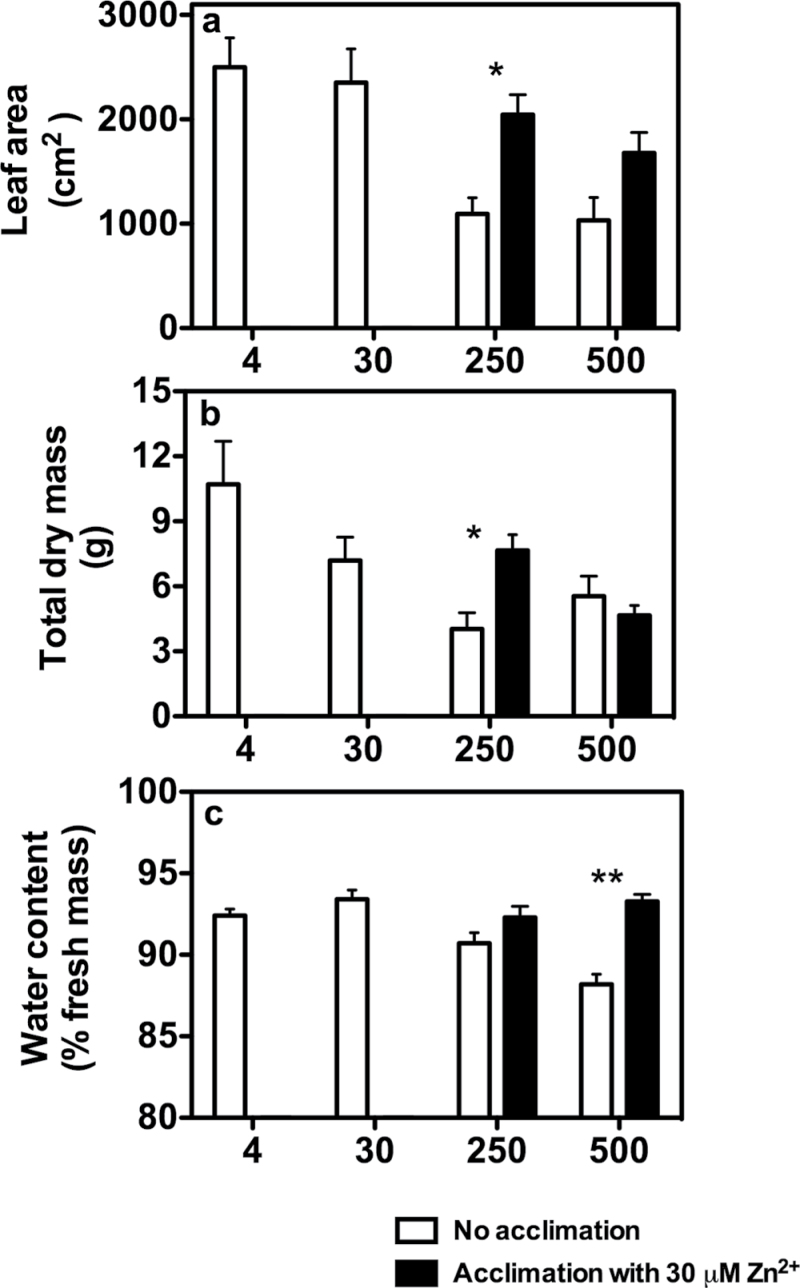
Response of tobacco plants to increasing concentration of ZnSO_4_ in the root zone. (a) Leaf area. (b) Total dry mass. (c) Plant WC. In six treatments, the plant root systems were exposed increasing Zn^2+^ concentrations (1, considered as the control treatment, 30, 250, and 500 μM ZnSO_4_). In the remaining two treatments, 1 week prior to the treatments (250 and 500 μM ZnSO_4_), plants were exposed to 30 μM ZnSO_4_. Values are mean±standard error (SE) (*n*=4). Asterisks indicate significant differences between acclimated and non-acclimated treatments. *P<0.05, **P<0.01.

**Fig. 2.**
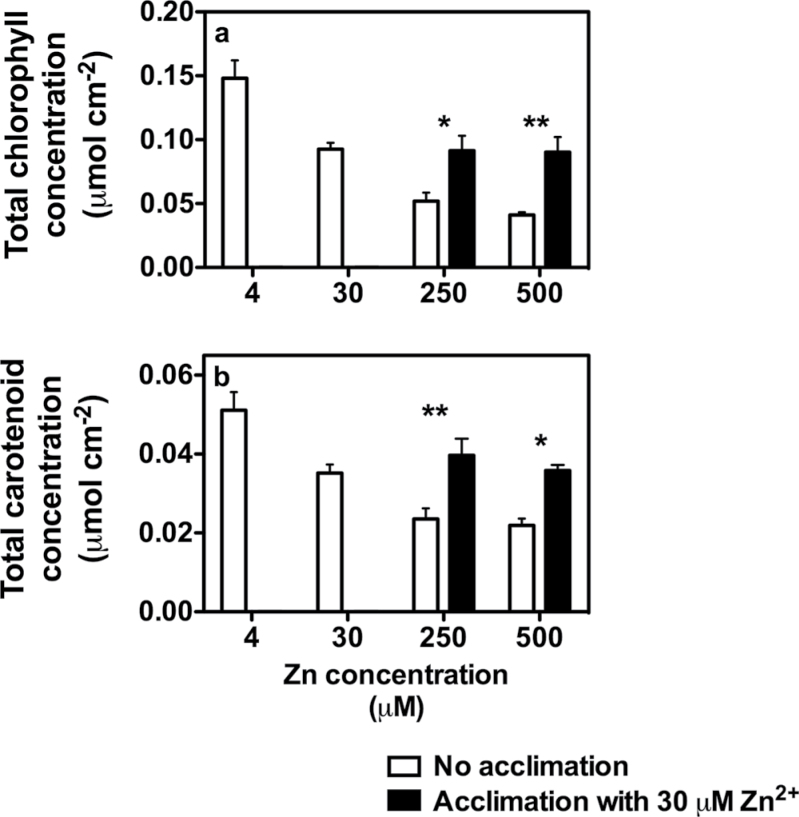
Total chlorophyll and carotenoid concentrations in tobacco plants in response to increasing ZnSO_4_ concentrations in the root zone. In six treatments, the plant root systems were exposed increasing Zn^2+^ concentrations (1, considered as the control treatment, 30, 250, 500 μM ZnSO_4_). In the remaining two treatments, 1 week prior to the treatments (250 and 500 μM ZnSO_4_) plants were exposed to 30 μM ZnSO_4_. Values are mean±SE (*n*=4). Asterisks indicate significant differences between acclimated and non-acclimated treatments. *P<0.05, **P<0.01.

After 3 weeks of Zn^2+^ treatments, leaf area and plant dry mass gradually declined with increasing Zn^2+^ concentrations in the root zone (Fig. 1a, b). With 30 μM ZnSO_4_, both the leaf area and the total dry mass remained similar to that in control plants; however, with 250 and 500 μM ZnSO_4_ both parameters substantial declined (≥50%). For example, with 250 and 500 μM ZnSO_4_, leaf area respectively declined by 52% and 78% compared with values in control plants (Fig. 1a). One week of acclimation reduced the toxic effect of Zn^2+^, mostly with 250 μM ZnSO_4_, as with 500 μM ZnSO_4_, dry mass was similar in acclimated and non-acclimated plants (Fig. 1b). Indeed, with 250 μM ZnSO_4_, both leaf area and total dry mass in acclimated plants increased by 60% compared with those in the corresponding non-acclimated 250 μM ZnSO_4_ treatment. Plant WC declined significantly (*P≤*0.05) with only 500 μM ZnSO_4_ (Fig. 1c), declining from 95% in control plants to 88% in non-acclimated plants exposed to 500 μM ZnSO_4_. Also for this parameter, the acclimation period had a positive effect, and plant WC in acclimated plants exposed to 500 μM ZnSO_4_ remained similar to that of control plants (Fig. 1c). Total chlorophyll and carotenoid concentrations declined with increasing Zn^2+^ concentration in the root medium, with marked declines at 250 and 500 μM ZnSO_4_. Compared with control plants, total chlorophyll concentration with 250 and 500 μM ZnSO_4_ was reduced by 65 and 72%, respectively (Fig. 2a); similarly, carotenoid concentrations in plants exposed to 250 and 500 μM ZnSO_4_ declined by 54–57% (Fig. 2b). As observed for leaf area and total dry mass, the acclimation period reduced the toxic effect of 250 and 500 μM ZnSO_4_, and compared with the values in non-acclimated plants, both total chlorophyll and carotenoids concentrations in acclimated plants increased by 40–90%.

### Acclimation with 30 µM ZnSO_4_ reduces root damage

**Fig. 3.**
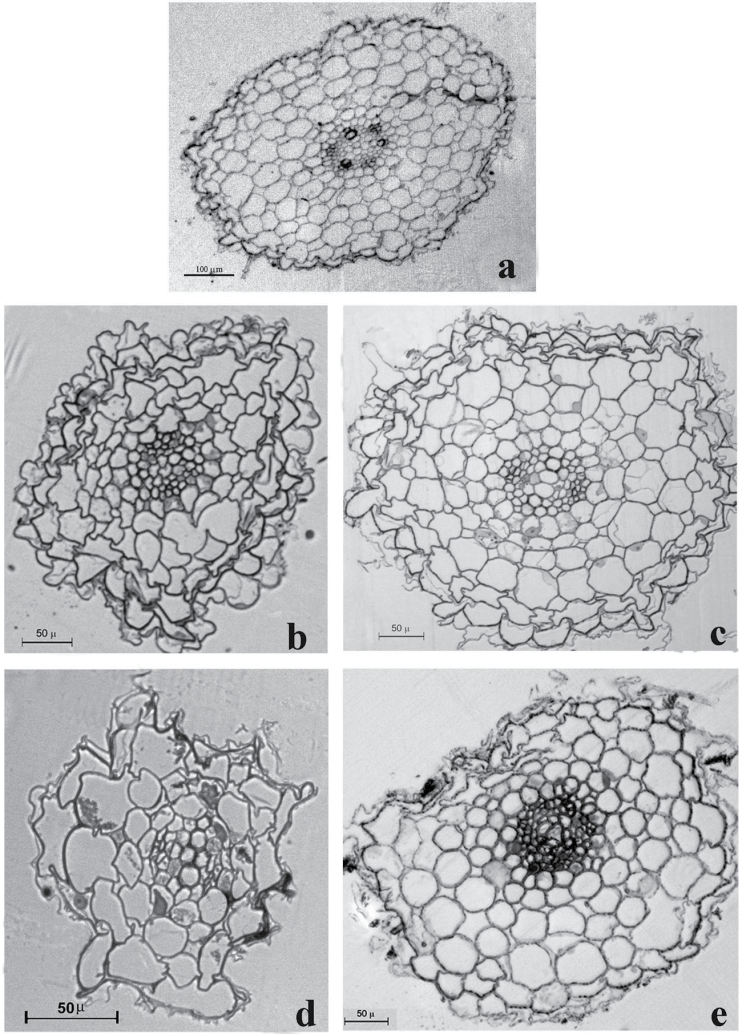
Transverse sections of roots of tobacco plants exposed to increasing concentration of ZnSO_4_ in the root zone. (a) Roots of plants grown in the control nutrient solution for 3 weeks. (b) Roots of plants grown with 250 μM ZnSO_4_ with no prior acclimation. (c) Roots of plants grown for 1 week with 30 μM ZnSO_4_ and then exposed to 250 μM ZnSO_4_. (d) Roots of plants grown with 500 μM ZnSO_4_ with no prior acclimation. (e) Roots of plants grown for 1 week with 30 μM ZnSO_4_ and then exposed to 500 μM ZnSO_4_.

**Fig. 4.**
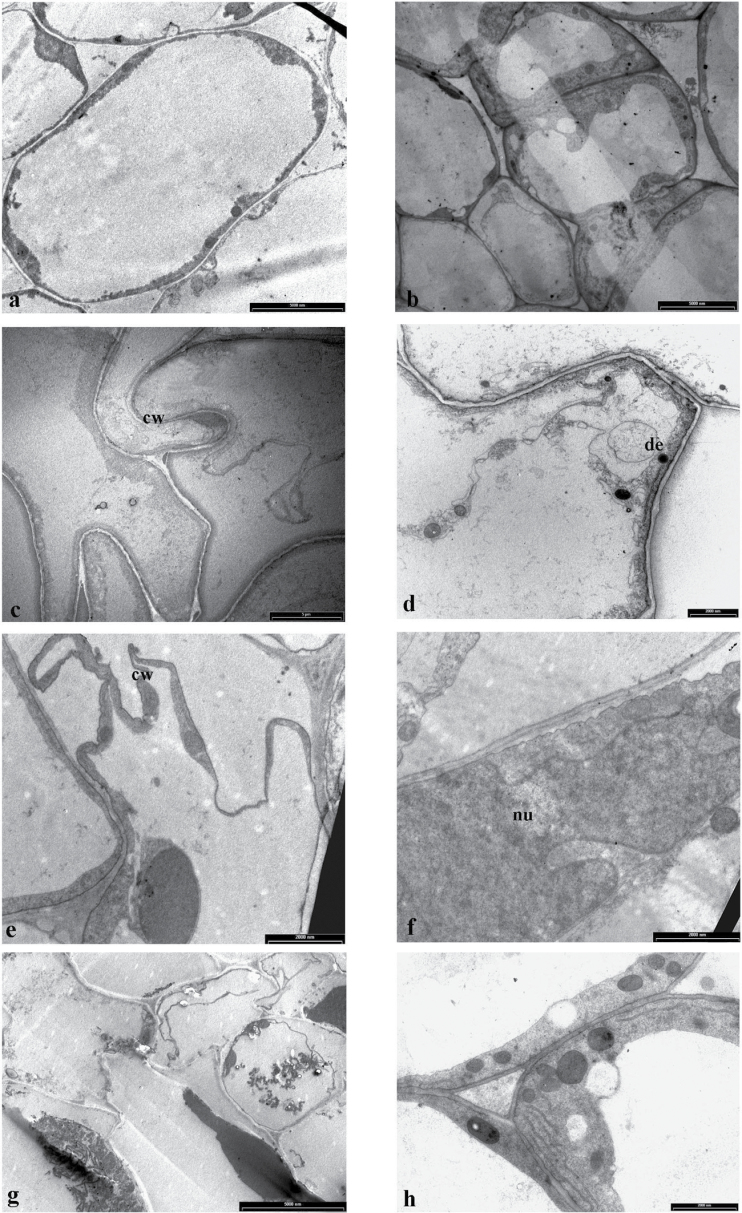
Electron micrographs of transverse section of root cells of tobacco plants exposed to increasing concentration of ZnSO_4_. (a, b) Plants grown in the control nutrient solution for 3 weeks showing a cortical cell (a) and central cylinder cells (b). (c, d) Cortical cells of plants grown with 250 μM ZnSO_4_ with no prior acclimation period showing a tortuous cell wall (c) and a damaged cell with deposits (d). (e, f) Plants grown for 1 week with 30 μM ZnSO_4_ and then exposed to 250 μM ZnSO_4_ showing irregular cell walls (e) and an irregular nucleus (f). (g) Plants grown with 500 μM ZnSO_4_, with no prior acclimation period, with disintegrated cytoplasmic organules in the cells of the central cylinder. (h) A cortical cell of plants grown for 1 week with 30 μM ZnSO_4_ and then exposed to 500 μM ZnSO_4_ with well-preserved cytoplasmic organules. cw, cell wall; de, deposit; nu, nucleus.

In control roots, the cortical cells showed large nuclei and large vacuoles, long endoplasmic reticulum, mitochondria with well-developed cristae and several proplastids with short lamellae. Cortical and central cylinder cells exhibited regular shape (Figs 3a and 4a, b). The presence of elevated Zn^2+^ in the root zone severely damaged roots. Plants treated with 250 μM ZnSO_4_ had the cells of the cortical layer damaged (Fig. 3b), with a tortuous cell wall (Fig. 4c); furthermore, some cortical cell had disintegrated cytoplasmic content with deposits in the cytoplasm (Fig. 4d). Damage was more evident with 500 μM ZnSO_4_; at this concentration, roots had the epidermis and most of the cortical cells completely destroyed (Fig. 3d), with disintegrated cytoplasmic content. Furthermore, plants exposed to 500 μM ZnSO_4_ had cells in the central cylinder with collapsed cytoplasmic organelles (Fig. 4g). The acclimation period with 30 μM ZnSO_4_ reduced root injuries in response to Zn^2+^ stress. Acclimated plants exposed to 250 μM ZnSO_4_ exhibited reduced damage in cortical cells compared with non-acclimated ones (Fig. 3c), with healthy cells in the central cylinder, although there were a few cortical cells that had nuclei and cell walls with an irregular shape (Fig. 4e, f). With 500 μM ZnSO_4_, although in acclimated plants the root epidermis (Fig. 3e) and several cortical cells were damaged, in the cortex there were cells with well-preserved cell organelles (Fig. 4h), and the central cylinder appeared perfectly functional, with cells showing all the cellular organelles.

### Zinc rapidly affects *g*_s_ in non-acclimated plants but not in those acclimated with 30 μM ZnSO_4_

To unravel the mechanism(s) responsible for improved tolerance observed in acclimated plants, two more experiments were conducted focusing on the 250 μM ZnSO_4_ treatment. To evaluate the effect of 250 μM ZnSO_4_ upon CO_2_ fixation with respect to leaf conductance, these two parameters were recorded before and after ZnSO_4_ application (Table 1). After 24h of treatment, although growth was not affected and there were no visible differences in plant WC (Supplementary Table S2 at *JXB* online), *A*_n_ declined by 18% in the leaves in non-acclimated plants compared with control plants (Table 1). By contrast, *A*_n_ in acclimated plants remained similar to that of control plants. The negative effects of 250 μM ZnSO_4_ in non-acclimated plants were, however, more evident in the greater inhibition of leaf *g*_s_. Indeed, compared with initial values, in plants without the acclimation period, the addition of 250 μM ZnSO_4_ decreased *g*_s_ by 40%, while in acclimated plant *g*_s_ remained similar to *g*_s_ in control plants (Table 1). Already after 24h of treatments, the stronger inhibition of *g*_s_ compared with *A*_n_ led to a visible (7%) reduction in the *C*_i_ in non-acclimated plants compared with C_i_ in acclimated plants (Table 1). It is unlikely that these reductions in *g*_s_ observed in non-acclimated plants in the first 24h of treatment were associated with specific Zn^2+^ toxicity at the leaf level; indeed, leaf Zn^2+^ concentrations in acclimated plants were several fold higher than those in non-acclimated plants, due to the 1-week exposure to Zn^2+^ prior to the treatment in acclimated plants, while non-acclimated plants were exposed to Zn^2+^ for only 24h (Supplementary Fig. S1 and Table S3 at *JXB* online). Chlorophyll fluorescence results showed that the maximum quantum efficiency of PSII was not affected by 250 μM ZnSO_4_, independently of the acclimation period (Table 1).

**Table 1.** Leaf photosynthetic rate (A_n_), stomatal conductance (g_s_), substomatal CO_2_ concentration (C_i_), and maximum quantum efficiency of PSII photochemistry (F_v_/F_m_) in leaves of tobacco plants 24h after the addition of 250 μM ZnSO_4_ in non-acclimated plants and in plants acclimated with 30 μM ZnSO_4_ for 1 week

Parameter	Treatment ZnSO_4_ (μM)			
	1	1–30	1–250	30–250
*A*_n_ (μmol m^–2^ s^–1^)	4.88±0.22^a,b^	4.50±0.22^a,b^	3.98±0.28^b^	4.95±0.18^a^
*g*_s_ (mmol m^–2^ s^–1^)	115±6^a^	102±0^a,b^	70±3^b^	121±12^a^
*C*_i_ (μmol m^–2^)	319±4^a^	314±3^a^	297±1^b^	312±4^a^
*F*_v_/*F*_m_	0.78±0.01^a^	0.78±0.00^a^	0.77±0.00^a^	0.77±0.01^a^

Data are mean±SE (*n*=4). Different superscript letters within a row indicate significant differences between treatments (*P*<0.05). Initial values (prior to the treatment) for control and acclimated plants were, respectively: *A*_n_, 4.9 ± 0.2 and 4.4 ± 0.2 μmol m^–2^ s^–1^; *g*_s_, 111 ± 2 and 119 ± 8 mmol m^–2^ s^–1^; *C*_i_, 312 ± 2 and 301 ± 5 μmol m^–3^.

### Acclimation with 30 μM ZnSO_4_ is associated with a more negative membrane potential in root cortical cells

**Fig. 5.**
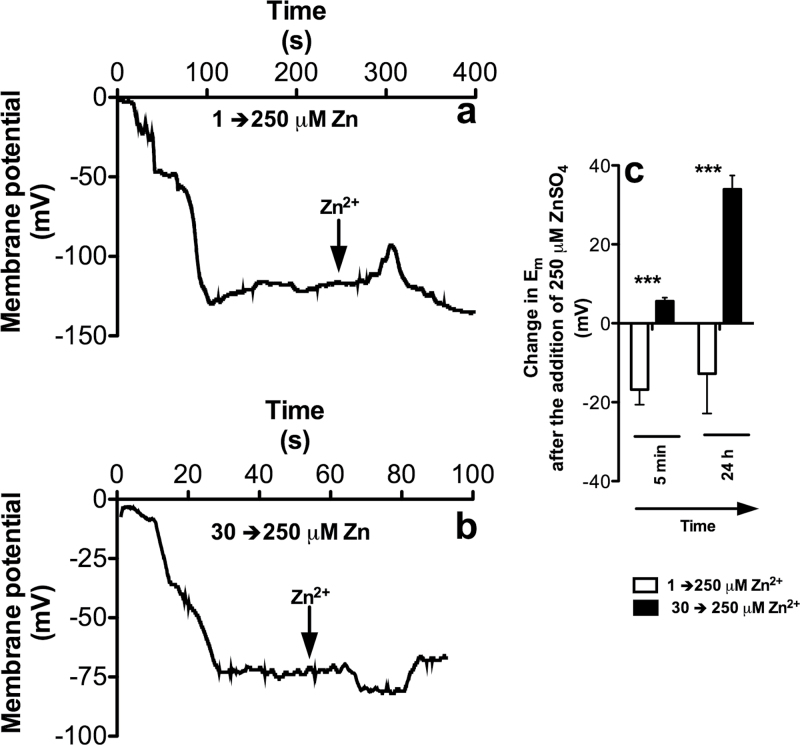
(a, b) Zn^2+^-dependent changes in *E*_M_ in root cortical cells of tobacco plants after the addition of ZnSO_4_ (with a final concentration of 250 μM ZnSO_4_ in the medium) in non-acclimated (a) and acclimated (b) plants. (c) Mean values±SE (*n*=15–20) of membrane depolarization and hyperpolarization in cortical root cells of non-acclimated and acclimated plants. Immediately after adding of ZnSO_4_ to the medium, transient depolarization occurred in non-acclimated plants (a, c). By contrast there was a transient hyperpolarization in plants acclimated with 30 μM ZnSO_4_ 1 week prior to the addition of ZnSO_4_, which lasted for the following 24h (b, c). Asterisks indicate significant differences between acclimated and non-acclimated treatment. ***P<0.001.

The effects of the addition of 250 μM ZnSO_4_ were monitored to determine whether early changes in the membrane potential (*E*_M_) of root cortical cells could explain the increased tolerance in acclimated plants. Immediately after adding ZnSO_4_ to the medium, the root cortical cells of non-acclimated plants transiently depolarized by 16.8±3.8 mV (Fig. 5a, c). By contrast, in acclimated plants, there was an opposite behaviour, and immediately after addition of the stress, there was a transient hyperpolarization of *E*_M_ (5.6±3.7 mV, Fig. 5b, c). We then assessed how *E*_M_ changed after 24h of exposure to 250 μM ZnSO_4_, and observed that there were substantial differences in the *E*_M_ of cortical cells in acclimated and non-acclimated plants. In the non-acclimated plants, parts of the plant were able to partially repolarize their plasma membrane, and thus after 24h of treatment there were no significant differences in *E*_M_ when compared with control plants. On the other hand, *E*_M_ in acclimated plants remained hyperpolarized, with values on average 37% more negative compared with those in non-acclimated plants exposed to 250 μM ZnSO_4_ (–91.2±5.3 mV in non-acclimated plants vs –125.1±3.5 mV in acclimated plants, Fig. 5c).

### Acclimation with 30 μM ZnSO_4_ enhances vacuolar Zn^2+^ sequestration in roots

**Fig. 6.**
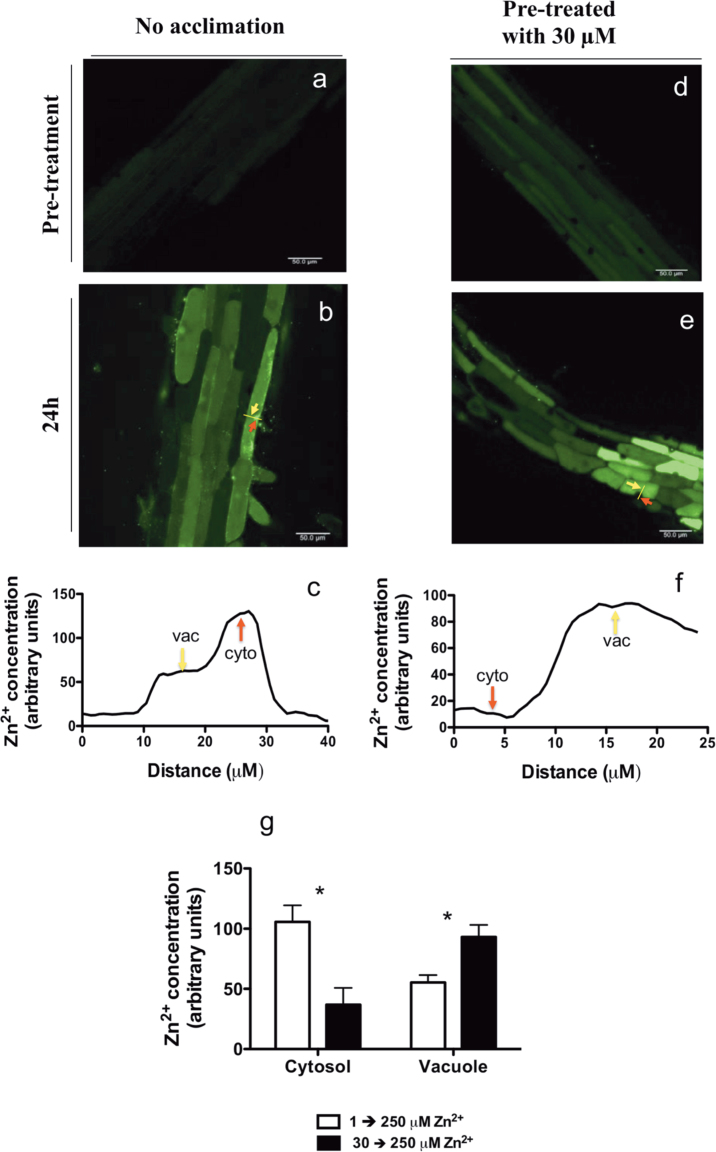
Zinc compartmentation in root epidermal cells of tobacco plants before (a, d) and after (b,e) the addition of ZnSO_4_ (with a final concentration of 250 μM ZnSO_4_ in the medium) in non-acclimated (a, b) and acclimated (d, e) plants. One typical root for non-acclimated (c) and non-acclimated (f) plants is shown. Measurements were made in the mature zone, between 10 and 20mm from the root apex. In (g), quantification of the cytosolic:vacuolar Zn^2+^ content ratio in epidermal root cells was evaluated and values are given as means±SE (*n*=12–16). The Zn^2+^ content in each cell compartment is proportional to the intensity of FluoZin-3-AM (showed in arbitrary units). Asterisks indicate significant differences between acclimated and non-acclimated treatment. *P<0.05.

The ability to compartmentalize Zn^2+^ in the cell vacuole provides an effective mechanism to avoid the toxic effects of Zn^2+^ in the cytoplasm (Hall, 2002; Krämer *et al.*, 2007). Given the striking differences in root membrane depolarization and hyperpolarization patterns between acclimated and non-acclimated plants, we investigated whether these different responses were associated with different abilities to compartmentalize Zn^2+^ in the roots. In order to evaluate it, confocal laser-scanning microscopy was used to observe the intracellular distribution of Zn^2+^ in root epidermal cells. After 24h of 250 μM ZnSO_4_, in non-acclimated plants, most of the accumulated Zn^2+^ was found to be located prevalently in the cytosol, with only a few cells showing good Zn^2+^ compartmentalization in the vacuole (Fig. 6a–c). By contrast, in acclimated plants, most of the Zn^2+^ was located in the vacuole, indicating an efficient compartmentalization of accumulated Zn^2+^ (Fig. 6d–f). The intracellular spatial distribution of Zn^2+^ within the epidermal cells was further quantified using ImageJ software (National Institutes of Health, Bethesda, MD, USA). In the present work, arbitrary but not absolute values for intracellular Zn^2+^ concentrations were used, because for comparative purposes this semi-quantitative method has been found previously to be perfectly valid (Cuin *et al.*, 2011). As illustrated in Fig. 6g, the cytosolic Zn^2+^ content in acclimated plants was found to be consistently lower (on average by 60%) compared with vacuolar Zn^2+^, while in non-acclimated plants, the opposite was observed, and the cytosolic Zn^2+^ content was found to be, on average, double that of the respective vacuole (Fig. 6g). Therefore, cytosolic:vacuolar Zn^2+^ content ratio ranged from 0.4 in acclimated plants to 1.9 in non-acclimated plants.

### The acclimation process enhances the expression levels of the tobacco orthologue of the *A. thaliana MTP1* gene in roots

**Fig. 7.**
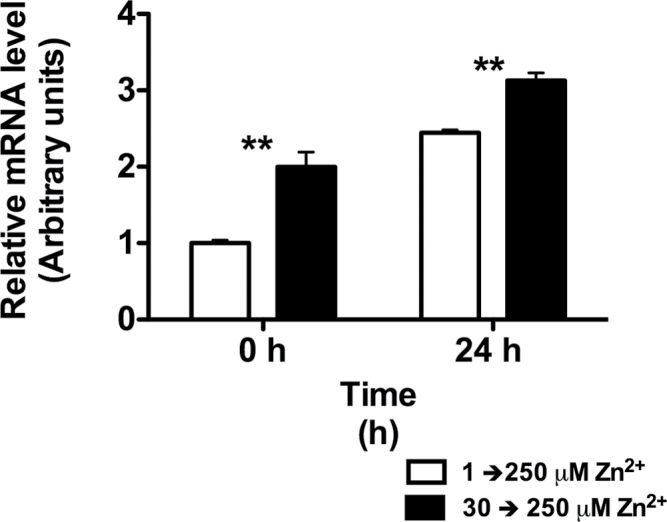
Relative transcript levels of the tobacco orthologue of the *A. thaliana MTP1* gene (*NtMTP1*) before (prior to the addition of Zn^2+^) and 24h after the addition of 250 μM ZnSO_4_ in the root zone. In two treatments, the plant root systems were exposed increasing Zn^2+^ concentrations (1, considered as the control treatment, and 250 μM ZnSO_4_). In the remaining treatment, 1 week prior to the treatment (250 μM ZnSO_4_), plants were exposed to 30 μM ZnSO_4_. Values are means±SE (*n*=4). The mRNA levels of genes for *NtMTP1* were determined by real-time PCR using specific primer pairs and normalized to that of the *EF 1-α*. Asterisks indicate significant differences between acclimated and non-acclimated treatment. **P<0.01.

Given that confocal data indicated that acclimated plants had a superior ability to efficiently sequester Zn^2+^ into root cell vacuoles, we measured the transcript levels of the tobacco orthologue of the *A. thaliana MTP1* gene (*NtMTP1*; for more details, see Shingu *et al.*, 2005). Indeed, in *A. thaliana*, *MTP* genes have been found to be involved in cellular detoxification and sequestration of Zn^2+^ in vacuoles, and thus directly linked to Zn^2+^ tolerance (Rascio and Navari-Izzo, 2011). The transcript levels of *NtMTP1* were found to vary in response to increasing Zn^2+^; thus, in acclimated plants already at time 0 (i.e. before adding the stress) transcript abundance (arbitrary units) was approximately 2-fold that in control plants (Fig. 7). The beneficial effects of the acclimation process were still evident following the addition of 250 μM ZnSO_4_; in acclimated plants, the transcript abundance further increased to approximately 3-fold the values in control plants whereas it only increased by approximately 1.5-fold in non-acclimated plants (Fig. 7).

## Discussion

Exposure to non-toxic Zn^2+^ concentrations resulted in an improved tolerance of tobacco plants to elevated Zn^2+^ concentrations. In non-acclimated plants, 250 and 500 μM ZnSO_4_ was highly toxic over the 3-week treatment period, with a marked decline (>50%) in plant growth. Conversely, a 1-week exposure to non-toxic Zn^2+^ concentrations prior to the Zn^2+^ treatments substantially reduced the Zn^2+^ toxic effects both at the root and at the shoot level; in acclimated plants, compared with non-acclimated ones, there was a 1.6-fold increase in plant dry mass and leaf area and up to a 2-fold increase in leaf pigment concentrations. Furthermore, at the root level, the anatomical analyses clearly showed that the roots of acclimated plants were less damaged by high Zn^2+^ concentrations. Indeed, although roots of acclimated plants showed ultrastructural alterations at the epidermal and cortical level, in both treatments roots presented a functioning central cylinder. By contrast, in non-acclimated plants directly exposed to 250 and 500 μM ZnSO_4_, roots showed more severe damage, with disruption of the epidermis and cortex, and in the case of 500 μM ZnSO_4_ also of the central cylinder. In order to elucidate the possible mechanism(s) responsible for the enhanced tolerance, short-term experiments were conducted focusing on the responses to 250 μM ZnSO_4_ in acclimated and non-acclimated plants. These experiments showed that the acclimation process induced specific detoxification mechanisms at the root level that, following the addition of elevated Zn^2+^ concentrations in the growth medium, reduced the build-up of Zn^2+^ in sensitive and metabolically active sites of the cell, ultimately resulting in an improved leaf stomatal regulation and an *E*_M_ hyperpolarization of the root cortical cells.

Root membrane potential data confirmed our hypothesis that the Zn^2+^ acclimation process led to an improved response at the root level, indicating an improved activity and regulation of plasma membrane-located processes. In non-acclimated plants, immediately after the roots were exposed to 250 μM ZnSO_4_, there was a rapid depolarization of the *E*_M_, with a partial repolarization of the plasma membrane 24h after the treatment. Interestingly, 1 week of acclimation with 30 μM ZnSO_4_ resulted in *E*_M_ hyperpolarization in root cortical cells, immediately after the addition of Zn^2+^ and for the following 24h. These results are in agreement with the recent finding of a clear link between *E*_M_ depolarization/hyperpolarization patterns in cortical root cells following Zn^2+^ addition (0.1–1mM) and the overall Zn^2+^ tolerance in three *Arabidopsis* species (Kenderesová *et al.*, 2012). In the above-mentioned study, the magnitude and duration of the Zn^2+^-dependent depolarization was higher in the sensitive *A. thaliana* than in the tolerant *Arabidopsis arenosa* and *Arabidopsis halleri*; the addition of 0.5mM ZnCl_2_ depolarized the root plasma membrane of *A. thaliana* by 26 mV, while in the hyperaccumulator *A. halleri* there was no significant depolarization.

The contrasting *E*_M_ depolarization/hyperpolarization patterns observed in the present study between control and acclimated plants were probably coupled with the detoxification of intracellular Zn^2+^. The intracellular distribution of Zn^2+^ in root epidermal cells of acclimated and non-acclimated plants supports the hypothesis that the acclimation period elicited specific detoxification mechanisms, i.e. enhanced vacuolar Zn^2+^ sequestration. Conversely, in non-acclimated plants, the addition of 250 μM ZnSO_4_ led to larger increases in cytosolic Zn^2+^ compared with vacuolar Zn^2+^. As a result, the cytosolic:vacuolar Zn^2+^ content ratio ranged from 0.4 in acclimated plants to 1.9 in non-acclimated plants, indicating a correlation between vacuolar Zn^2+^ sequestration and improved membrane functionality following Zn^2+^ exposure. Indeed compartmentation of metals within the cell and sequestration in the vacuoles is an effective way to maintain cytoplasmic Zn^2+^ concentrations as low as necessary, keeping toxic Zn^2+^ away from active cellular metabolic components (Krämer *et al.*, 2007).

Maintenance of metal homeostasis in cells and their transport across the plasma membrane, tonoplast, and other endomembranes is achieved by the activity of specific transporters and metal pumps. Interestingly, in parallel with the increases in Zn^2+^ vacuolar sequestration, we also observed that the acclimation period increased the transcript levels of the tobacco orthologue of the *A. thaliana MTP1* gene in roots. Despite this gene having yet to be characterized in tobacco, in *A. thaliana* vacuolar *MTP* has been shown to be involved in the active transport of Zn^2+^ from the cytosol into the vacuole (Kobae *et al.*, 2004; Arrivault *et al.*, 2006; Gustin *et al.*, 2009; Kawachi *et al.*, 2009). In the present study, the expression levels of *NtMPT1* increased following Zn^2+^ exposure, in a dose-dependent manner (Fig. 7), which contrasts with several published studies that clearly show that expression of *MTP1* in *A. thaliana* is not induced by Zn^2+^ exposure. However, given that expression levels of *MTP3* have been shown to increase following zinc exposure (Kobae *et al.*, 2004; Arrivault *et al.*, 2006; Gustin *et al.*, 2009), we performed a protein sequence analysis using the Conserved Domain Database (http://www.ncbi.nlm.nih.gov/Structure/cdd/cdd.shtml). From this analysis, it emerged that the functional domains of *NtMTP1*, *AtMTP1*, and *AtMTP3* are conserved and that these proteins are characterized by a histidine-rich domain, from aa 182 to 232 (Supplementary Fig. S2 at *JXB* online); this histidine-rich domain has been shown to have a regulatory function on the activity of the protein (Kawachi *et al.*, 2008). Interestingly, sequence alignments of *MTP* in *A. thaliana* and tobacco highlighted a high similarity between the histidine-rich domain in *NtMTP1* and *AtMTP3* (Supplementary Fig. S2); it is therefore plausible that, despite the *NtMTP1* sequence being closer to that of *AtMTP1*, the function of the protein in tobacco may indeed be more similar to that of *AtMTP3*, thus explaining the observed results. However, this statement remains speculative, and further studies are clearly needed to validate this hypothesis. Furthermore, given that 24h of exposure to 30 μM ZnSO_4_ was not sufficient to induce detectable increases in *NtMTP1* transcript levels, it would be reasonable to expect that a threshold/minimum acclimation period is required for the pre-exposure to non-toxic levels of Zn^2+^ to induce acclimation in roots. A similar time- and concentration-dependent increase in *MTP3* transcript levels has been reported previously in *A. thaliana* roots exposed to different Zn^2+^ concentrations, with higher transcript levels at higher Zn^2+^ concentrations and an almost linear increase in the pMTP3::GUS activity during the first 8 d of exposure to 30 μM ZnSO_4_ (Arrivault *et al.*, 2006). The hypothesis that there is a minimum time required, depending on the Zn^2+^ concentration used, to induce acclimation would explain the contradictory results found in the literature regarding plant acclimation to heavy-metal stress (e.g. Turner and Dickinson 1993; Wisniewski and Dickinson 2003).

Elevated Zn^2+^ concentrations in the root zone increased the resistance of the CO_2_ pathway from the atmosphere to the sites of carboxylation. Gas-exchange data suggested that Zn^2+^ treatments, over the 24h treatment period, mainly affected stomatal functioning rather than the photosynthetic machinery. Indeed, if the increase in stomatal limitation is the dominant cause of the reduction in *A*_n_ (Fig. 2c), then *C*_i_ must decrease (Long and Hallgren, 1985; Bednarz *et al.*, 1998). Therefore, given that in non-acclimated plants the declines in *g*_s_ were paralleled by declines in *A*_n_ and *C*_i_, these results would support the view that photosynthesis was limited by the low leaf conductance resulting from stomatal closure (Medrano *et al.*, 2002). Accordingly, when *Beta vulgaris* was grown with 100 and 300 μM Zn^2+^, stomatal limitations accounted for 79–86% of the total photosynthesis reduction, whereas mesophyll conductance accounted only for the remaining 14–21%, and non-significant biochemical limitations occurred. Several factors could explain the declines in stomatal opening in non-acclimated plants. Given that leaf Zn^2+^ concentrations in acclimated plants were several fold higher than those in non-acclimated plants (Supplementary Fig. S1 and Table S3), it would appear that the higher *g*_s_ in acclimated plants, rather than being associated with an improved detoxification processes, could have been dependent on the improved root plasma-membrane functionality. Disturbed root membrane functionality in the long term may result in increased lipid peroxidation and increased membrane permeability, which in turn increase plant water losses or increase membrane resistance, thus reducing water uptake (Barceló and Poschenrieder, 1990; Kamaluddin and Zwiazek, 2004; Llamas *et al.*, 2008). In a recent study, it was found that heavy metals very quickly (within the first few minutes after the application) reduced overall water permeability of the epidermal cells of *Allium cepa* bulb (Przedpelska-Wasowicz and Wierzbicka, 2011). These changes in water permeability in response to heavy metals can be caused by aquaporin gating, key proteins involved in regulating water flow across membranes, and/or by a general failure of cell metabolism (including aquaporin activity) due to heavy-metal toxicity (Przedpelska-Wasowicz and Wierzbicka, 2011). It is therefore conceivable that, in acclimated plants, the improved plasma-membrane functionality at the root level following Zn^2+^ stress avoided or reduced the inhibition of water flux across membranes, thereby explaining the improved stomatal regulation in acclimated plants.

In conclusion, elevated concentrations of heavy metals can have detrimental effects on plants at the cellular and whole-plant level. In the present study, we showed that the acclimation process was dependent on an improved response to elevated Zn^2+^ concentrations at the root plasma-membrane level, which in turn enhanced shoot performance. This improved functionality of the root plasma membrane in acclimated plants was dependent on the translocation of metal ions towards the vacuoles into metabolically inactive compartments (e.g. vacuole), thus avoiding toxic concentrations of metal in sensitive and metabolically active sites of the cell (Hall, 2002; Krämer *et al.*, 2007).

## Supplementary data

Supplementary data are available at *JXB* online.

Supplementary Table S1. PCR primers used in this study.

Supplementary Table S2. Shoot dry mass, root dry mass, shoot, and root WC in tobacco plants exposed to different concentrations of ZnSO_4_ in the root zone for 24h.

Supplementary Table S3. *NtMTP1* relative transcript levels and Zn^2+^ concentrations in young fully expanded leaves of tobacco plants exposed to different concentrations of ZnSO_4_ in the root zone for 24h.

Supplementary Fig. S1. Zinc compartmentation in cells of tobacco leaves exposed to different concentrations of ZnSO_4_ in the root zone for 24h.

Supplementary Fig. S2. Alignment of the histidine rich domain of *NtMTP1*, *AtMTP1* and *AtMTP3*.

Supplementary Data

## Abbreviations:

*A*_n_: net photosynthetic rate
*C*_i_: substomatal CO_2_ concentrations
*g*_s_: stomatal conductance
MTP: metal tolerance protein
PSII: photosystem II
RT-qPCR: quantitative real-time PCR
WC: water content.
