# Supplementary material for: Zn2+-induced changes at the root level account for the increased tolerance of acclimated tobacco plants
Source: J Exp Bot. 2014 Jun 13;65(17):4931–42. doi: 10.1093/jxb/eru251 (PMC4144771; doi:10.1093/jxb/eru251)
Supplement: Supplementary Data [file supp_65_17_4931__index.html]

Zn2+-induced changes at the root level account for the increased tolerance of acclimated tobacco plants — Zn2+-induced changes at the root level account for the increased tolerance of acclimated tobacco plants — Supplementary Data 

# Zn2+-induced changes at the root level account for the increased tolerance of acclimated tobacco plants

## Supplementary Data

Data files

**Files in this Data Supplement:**

- Supplementary Data - Supplementary Data
