# Supplementary material for: Zn2+-induced changes at the root level account for the increased tolerance of acclimated tobacco plants
Source: J Exp Bot. 2014 Jun 13;65(17):4931–42. doi: 10.1093/jxb/eru251 (PMC4144771; doi:10.1093/jxb/eru251)
Supplement: Supplementary Data [file supp_eru251_jexbot124784_file001.pdf]

Supplementary material:

Zn^{2+}-induced changes at the root level account for the increased zinc tolerance of acclimated tobacco plants

Nadia Bazihizina[1], Cosimo Taiti[1], Lucia Marti[1], Ana Rodrigo-Moreno[1], Francesco Spinelli[1], Christiana Giordano[2], Stefania Caparrotta[1], Massimo Gori[1], Elisa Azzarello[1], Stefano Mancuso[1]

[1] LINV - Department of Agrifood Production and Environmental Sciences – University of Florence, Viale delle Idee 30, 50019 Sesto F.no, Florence, Italy

[2] Centro di Microscopie Elettroniche " Laura Bonzi" (Ce.M.E.), ICCOM, CNR, Via Madonna del Piano, 50019 Sesto F.no, Florence, Italy

[*]To whom correspondence should be addressed. E-mail: stefano.mancuso@unifi.it

Tables

Table S1. PCR primers used in this study

[illegible]fSeq	size pb	Primer name	Fw 5'-------à3'	Rev 5'-------à3'
[illegible]201240	11	MTP111	tgtcgaggttgttggtggta	gcccagagtgaaaacaagga
[illegible]120093	67	EF 1alfa 67	tgtgacttttggtcccactg	aagagcttcgtggtgcatct
[illegible]18908	50	L25 50	CCCCTCACCACAGAGTCTGC	AAGGGTGTTGTTGTCCTCAATCTT
[illegible]026056	55	Ntubc2 55	ctggacagcagactgacatc	CAGGATAATTTGCTGTAACAGATTA

Table S2. Shoot dry mass, root dry mass, shoot, and root water content in tobacco plants exposed to different concentrations of $ZnSO_4$ in the root-zone for 24 h. In two treatments, the plant root systems were exposed to 1 (considered as the control treatment) and 250 μM $ZnSO_4$. In the remaining treatment, 1 week prior to the treatment (250 μM $ZnSO_4$) plants were exposed to 30 μM $ZnSO_4$. Values are mean ± SE (n = 4). Different upper case letters indicate significant differences at each time ($P < 0.05$).

Parameter	Treatment $ZnSO_4$ (μM)		
	**1**	**1 ➔ 250**	**30 ➔ 250**
**Shoot dry mass (g)**	0.183 ± 0.011[a]	0.190 ± 0.052[a]	0.197 ± 0.006[a]
**Root dry mass (g)**	0.020 ± 0.000[a]	0.019 ± 0.009[a]	0.027 ± 0.008[a]
**Shoot water content (% fresh mass)**	95.9 ± 0.1[a]	96.1 ± 0.2[a]	96.2 ± 0.1[a]
**Root water content (% fresh mass)**	97.0 ± 0.3[a]	97.2 ± 0.2[a]	96.0 ± 0.5[a]

Table S3. *NtMTP1* relative transcript levels and Zn^{2+} concentrations in young fully expanded leaves of tobacco plants exposed to different concentrations of $ZnSO_4$ in the root-zone for 24 h. In two treatments, the roots were exposed to 1 (considered as the control treatment) and 250 µM $ZnSO_4$. In the remaining treatment, 1 week prior to the treatment (250 µM $ZnSO_4$) plants were exposed to 30 µM $ZnSO_4$. Values are mean ± SE (n = 4). Different upper case letters indicate significant differences between treatments.

**Parameter**	**Treatment $ZnSO_4$ (µM)**		
	**1**	**1 ➔ 250**	**30 ➔ 250**
**Relative mRNA levels of *NtMTP1* (Arbitrary units)**	1.00 ± 0.05[a,b]	1.29 ± 0.09[b]	0.98 ± 0.05[a,b]
**Zinc concentration (mg g^{-1} DM)**	0.040 ± 0.004[a]	0.097 ± 0.005[b]	1.152 ± 0.129[c]

In order to determine whether differences in leaf gas exchange parameters after 24 h were triggered by with changes in ion concentrations, a representative sample of ground shoot tissues in Expt 2 was taken to determine tissue ion concentrations. Zn^{2+} concentrations were determined after digesting plant tissues in a mixture of concentrated HNO_3 and $HClO_4$ (2:1 v/v, Sigma–Aldrich, Italy) using a digester (VELP Scientifica, Italy). After digestion, the ion content was measured by an inductively coupled plasma-optical emission spectrometer (ICP-OES, OPTIMA 2000 DV, PerkinElmer, USA). The ICP analytical standards (AA/ICP calibration/check standards for environmental analysis, 1 g L^{-1}) for the ions were supplied by Sigma–Aldrich (Italy).

Figures

Figure S1. Zinc compartmentation in cells of tobacco leaves exposed to different concentrations of $ZnSO_4$ in the root-zone for 24 h. In two treatments, the roots were exposed to 1 (considered as the control treatment) and 250 µM $ZnSO_4$. In the remaining treatment, 1 week prior to the treatment (250 µM $ZnSO_4$) plants were exposed to 30 µM $ZnSO_4$.

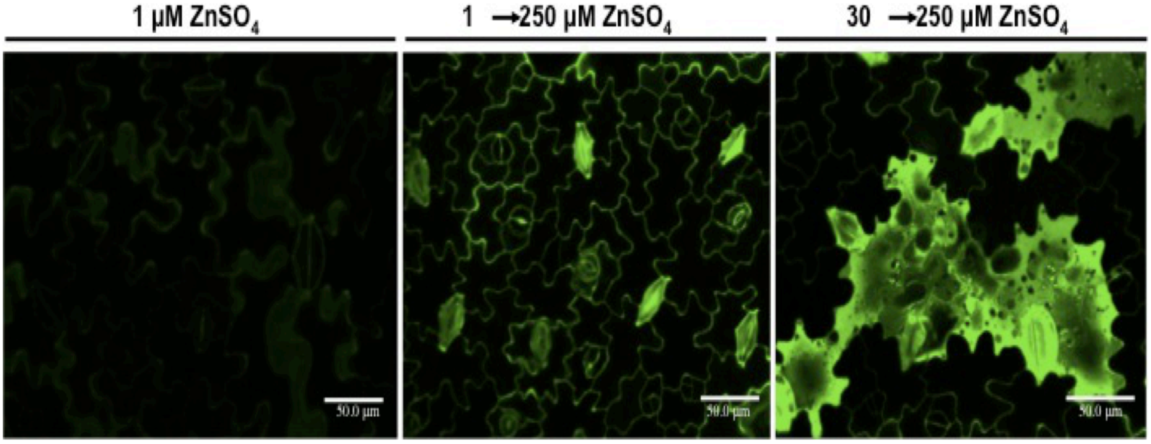

Figure S2. Alignment of the histidine rich domain of *NtMTP1*, *AtMTP1* and *AtMTP3*. The red line indicates the histidine rich domain.

```
NtMTP1    HDHGHGHGHSHGHDHGHEHGHNHEEHAHSHSDHEHGHGEH    218
AtMTP1    HDHGHSHGHGHG......................HGHDHH    199
Atmtp3    HDHGHGHGHSHDNGHGHSHDHGHGIAATEHHHDSGHDESQ    276

NtMTP1    THIHGISVSRHHHHNEGPSSRDQHSHAHDADHTEPLLKNS    258
AtMTP1    NHSHGVTVTTHHHHHDHEHGHSHGHGEDKHHAHGDVTEQL    239
Atmtp3    LSDVLIEQ................................    284

NtMTP1    CDGEGVPEGEKK                                270
AtMTP1    LDKSKTQVAAKE                                251
Atmtp3    ............                                284
```
